# Non-Steroidal Anti-Inflammatory Drugs Administered Intra-Articularly in Temporomandibular Joint Disorders: A Systematic Review and Meta-Analysis

**DOI:** 10.3390/jcm13144056

**Published:** 2024-07-11

**Authors:** Filip Bliźniak, Maciej Chęciński, Kamila Chęcińska, Karolina Lubecka, Monika Kamińska, Mariusz Szuta, Dariusz Chlubek, Maciej Sikora

**Affiliations:** 1 Department of Oral Surgery, Preventive Medicine Center, Komorowskiego 12, 30-106 Kraków, Poland; fblizniak@gmail.com (F.B.); maciej@checinscy.pl (M.C.); lubeckarolina@gmail.com (K.L.); 2 Department of Glass Technology and Amorphous Coatings, Faculty of Materials Science and Ceramics, AGH University of Science and Technology, Mickiewicza 30, 30-059 Kraków, Poland; checinska@agh.edu.pl; 3 Provincial Hospital in Kielce, ul. Grunwaldzka 45, 25-736 Kielce, Poland; lekmonikamk@gmail.com; 4 Department of Oral Surgery, Medical College, Jagiellonian University, Montelupich 4, 31-155 Kraków, Poland; m.szuta@wp.pl; 5 Department of Biochemistry and Medical Chemistry, Pomeranian Medical University, Powstańców Wielkopolskich 72, 70-111 Szczecin, Poland; sikora-maciej@wp.pl; 6 Department of Maxillofacial Surgery, Hospital of the Ministry of Interior, Wojska Polskiego 51, 25-375 Kielce, Poland

**Keywords:** diclofenac, intra-articular injections, non-steroidal anti-inflammatory drugs, NSAID, piroxicam, temporomandibular disorders, temporomandibular joint, tenoxicam

## Abstract

**Objectives**: This systematic review was designed to summarize randomized controlled trials of intra-articular administration of non-steroidal anti-inflammatory drugs (NSAIDs) for temporomandibular disorders. **Methods**: Randomized controlled trials regarding intra-articular injections of non-steroidal anti-inflammatory drugs for temporomandibular disorders were included in the review. The final search was conducted on 16 June 2024 in the Bielefeld Academic Search Engine, PubMed, and Scopus databases. **Results**: Of the 173 identified studies, 6 were eligible for review. In trials comparing arthrocentesis alone to arthrocentesis with NSAIDs, slight differences in joint pain were noted. For tenoxicam, differences were under 1 point on a 0–10 scale after 4 weeks, with inconsistent results. Piroxicam showed no significant difference, and pain levels were minimal in both groups. For maximum mouth opening (MMO), tenoxicam showed no significant difference. Piroxicam increased MMO by nearly 5 mm, based on one small trial with bias concerns. **Conclusions**: Currently, there is no strong scientific evidence supporting the injection of NSAIDs into the temporomandibular joint to relieve pain or increase jaw movement. Preliminary reports on piroxicam with arthrocentesis and tenoxicam or diclofenac without rinsing justify further research.

## 1. Introduction

### 1.1. Background

Non-steroidal anti-inflammatory drugs (NSAIDs) are medicines groups that have analgesic, anti-inflammatory, and antipyretic properties [1]. Their therapeutic effect as well as the risk of side effects result from the inhibition of cyclooxygenase 1 (COX-1) and cyclooxygenase 2 (COX-2) [2]. COX-1 is constantly present in the human body, and its action involves the synthesis of compounds such as prostaglandins and thromboxane, which play several important roles in the organism [3]. Prostaglandins are responsible, among other things, for creating the protective barrier of the stomach by reducing the production of hydrochloric acid and stimulating gastric mucus secretion [4]. Therefore, inhibiting the production of prostaglandins promotes the development of erosions in the digestive tract and the risk of bleeding [4]. The role of thromboxane is mainly vasoconstriction and platelet aggregation; thus, coagulation disorders may be a side effect of using NSAIDs [5]. The increased production of COX-2 occurs as a result of inflammation and its activation causes the symptoms that characterize it, such as redness, swelling, pain, increased temperature, and tissue function disorders. Therefore, NSAIDs may soothe the ailments mentioned above [6].

**Figure 1:**
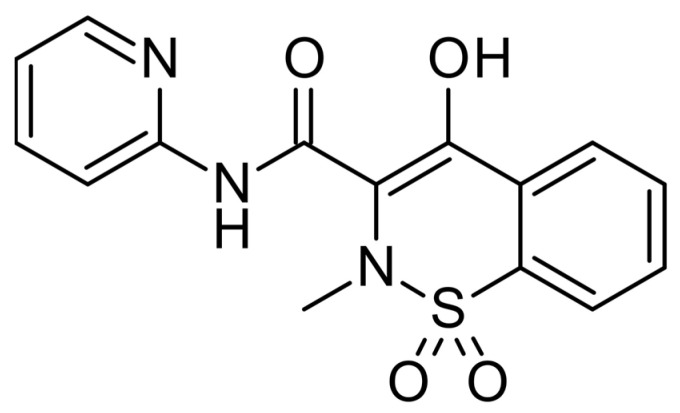
Piroxicam—chemical structure. Author: Fuse809, Public domain, via Wikimedia Commons.

**Figure 2:**
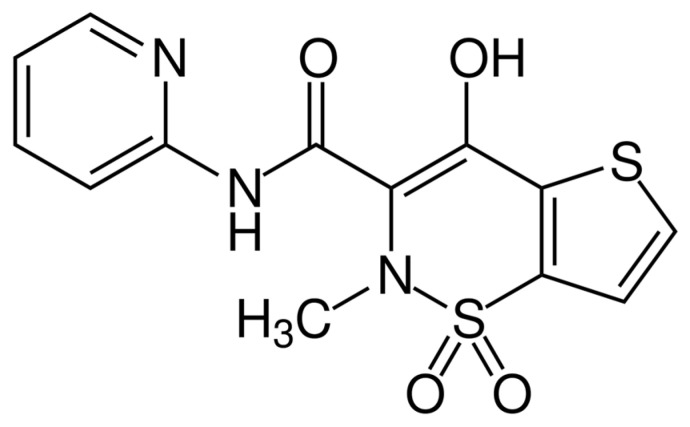
Tenoxicam—chemical structure. Author: Jü, Public domain, via Wikimedia Commons.

**Figure 3:**
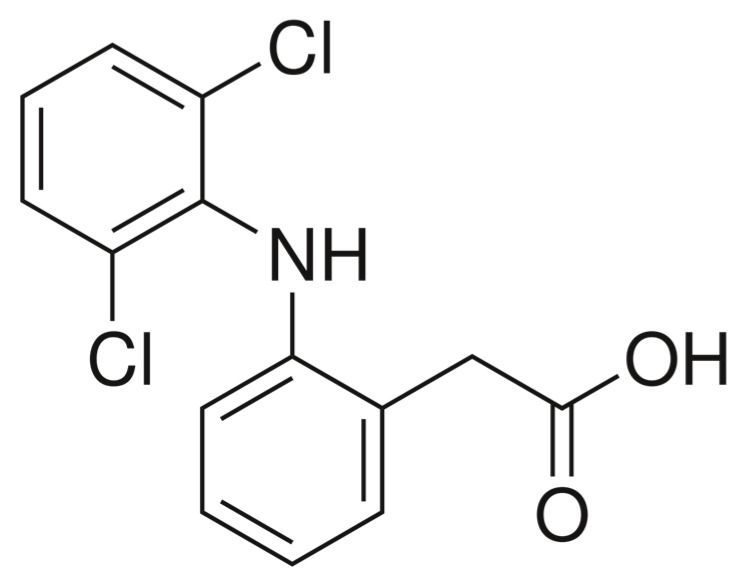
Diclofenac—chemical structure. Author: Harbin, Public domain, via Wikimedia Commons.

NSAIDs are a large group of drugs classified primarily due to their effect on cyclooxygenases: (1) inhibiting both COX-1 and COX-2 or (2) suppressing primarily COX-2 [7]. Preparations that inhibit COX-1 and COX-2 include, among others, ketoprofen, acetylsalicylic acid, indomethacin, nabumetone, ibuprofen, piroxicam (Figure 1), tenoxicam (Figure 2), meloxicam, diclofenac (Figure 3), naproxen, and nimesulide [8,9]. In turn, drugs that selectively suppress COX-2 are primarily coxibs [10]. These drugs can be administered orally (e.g., in the form of tablets, sachets, and syrups), rectally (suppositories), intravenously, intramuscularly, and in the form of creams, gels, and patches [11,12]. Depending on the classification of NSAIDs, their doses, or the dosing method, they exert both therapeutic effects and potential side effects with varying intensity [13].

Joint pain may be related to overload, or injury, but also result from the existence of a rheumatological disease, such as rheumatoid arthritis, systemic sclerosis, juvenile idiopathic arthritis, dermatomyositis, polymyositis, and systemic lupus erythematosus [14,15,16]. These diseases are among the diseases with chronic tissue inflammation and emerging pain [17,18]. In intra-articular injection therapy, mainly blood products, hyaluronic acid, and drugs are used [19,20]. The choice of NSAIDs potentially should reduce the exacerbation of inflammation and, consequently, relieve pain, which is one of its determinants. Scientific research has already addressed the issue of the intra-articular administration of NSAIDs, e.g., to the knee or hip joints. The indications for such treatment are relieving symptoms in orthopedic and rheumatological diseases and preventing complications associated with other administration routes [21].

Temporomandibular disorders (TMDs) manifest themselves, among others, with pain, limited mobility, and acoustic symptoms in the temporomandibular joints (TMJs) [22,23]. Secondary TMJ pain is attributed to arthritis, disc displacement, osteoarthritis, or subluxation. This paper focuses on the first three causes because subluxation is treated using other means, with particular attention to reducing jaw abduction and reducing the frequency of dislocation episodes. Recent reports indicate that TMDs affect between 5% and 12% of the population [24]. They also present a gender predilection, affecting women on average four times more often than men, although the reason for this has not yet been clearly established [25]. Based on research conducted among the population of the north-east of England, the cost of TMD treatment for a 6-month period is between EUR 379 and EUR 613 per person, which are mainly the costs of specialist consultations [26].

Several methods lead to relieve symptoms of TMDs, including physiotherapy, pharmacotherapy, splint therapy, and surgery [20,27,28]. The latter have a wide range of invasiveness, from single injections, through a series of administrations, two-needle arthrocentesis, and arthroscopy, to open surgery and even joint replacement with a prosthesis [29,30,31]. Intra-articular injections, as a minimally invasive surgical procedure combining the mechanical action of the administered fluid with the pharmacological action of the substance contained in it, have been the subject of many clinical studies summarized in systematic reviews [21,32]. Substances less frequently represented in the literature are still awaiting the synthesis of primary research results.

### 1.2. Rationale

There are increasing scientific articles on NSAIDs administered into the TMJs via injection [20]. Initial searches revealed that reports from clinical studies support the validity of such a procedure. However, the results of treatment with intra-articular NSAIDs require verification at a level higher than a single trial. The preliminary searches did not identify any systematic review specifically aimed at assessing the effectiveness of NSAID administration in the TMJ cavities. Broader reviews published to date only briefly address the topic of NSAIDs [20,32].

### 1.3. Objective

This systematic review aims to synthesize the randomized controlled trial evidence on NSAID intra-articular administration into the TMJ cavity.

## 2. Materials and Methods

This systematic review was conducted following the PRISMA 2020 [33] guidelines and registered to PROSPERO (CRD42024557962).

### 2.1. Eligibility Criteria

The systematic review included randomized trials on patients diagnosed with temporomandibular disorders. A necessary condition was the treatment of TMJ or TMJs with intra-articular NSAID administration. Co-interventions of lower invasiveness, e.g., physiotherapy, and the same invasiveness, e.g., arthrocentesis, were allowed. Protocols involving more invasive interventions, i.e., arthroscopic or open surgery, were excluded. The exclusion resulted from the assumption that the impact of the surgical procedure was more significant than the intra-articular deposition of NSAIDs. It was decided to evaluate the results in 3 categories: quality of life, pain intensity, and mandibular mobility. Due to the desire to conduct a review with the highest evidence, only randomized controlled trials were allowed. Reports not available as a full text in English were excluded (Table 1).

### 2.2. Information Sources and Search Strategy

The Bielefeld Academic Search Engine (BASE), PubMed, and Scopus were used to search medical article databases. Final searches were conducted on 16 June 2024. A follow-up search of the Cochrane Central Register of Controlled Trials was conducted on 4 July 2024.

The search strategy was based on a keyword constituting an essential element of diagnosis (“temporomandibular”) and two sets of keywords defining the intervention. These were a set of keywords for substances from the NSAID group and a set of keywords for the route of administration. Logical alternatives are used within the sets. The single keyword for diagnosis and two keyword sets for intervention were combined conjunctively. The following query was applied to all search engines:

temporomandibular AND (nsaid OR “non-steroidal anti-inflammatory drug*” OR salicylate OR *profen OR *fenac OR *oxicam OR piroxicam OR ampiroxicam OR meloxicam OR tenoxicam OR droxicam OR lornoxicam OR isoxicam OR phenylbutazone OR *fenamic OR fenamate OR *coxib OR nimesulide) AND (intra-articular OR intraarticular OR intra-cavitary OR intracavitary OR injection OR deposition)

No filters available in search engines were used. Search results were exported to source files for citation editors.

### 2.3. Selection Process

The identified records were entered into the Rayyan automation tool (version 2024-04-18, Qatar Computing Research Institute, Doha, Qatar and Rayyan Systems, Cambridge, MA, USA). Manual deduplication (M.C.) was then performed. In the next stage, two researchers conducted a blind selection of records based on titles and abstracts (K.L. and F.B.). In the case of inconsistency between the judges’ decisions, we promoted the record to the next stage. Cohen’s kappa value expressed the inter-rater agreement (version 22.030, MedCalc Software Ltd., Ostend, Belgium). A full-text assessment was then performed (K.L. and F.B.), where discrepancies were resolved by consensus, with a casting vote of a third researcher (M.C.) if necessary.

### 2.4. Data Collection Process

Data extraction was performed by a pair of authors (K.L. and F.B.). In the case of discrepancies in the interpretation of the report content or inconsistency of the collected numerical data, the decision was made by consensus, if necessary with a third vote from another author (M.C.). No automation tools were used. Numerical data were tabulated using the Google Workspace package (version 2024-04-17, Google LLC, Mountain View, CA, USA).

### 2.5. Data Items

Any quality of life questionnaire was accepted. Pain intensity was assessed only concerning joint pain. When different measurements were available, spontaneous pain intensity scores were preferred. The available data were transformed to a scale of 0–10, regardless of whether they were collected visually or numerically. In the assessment of mandibular mobility, maximum voluntary opening was preferred. In the absence of data, any other type of measurement of vertical mandibular mobility was used. This variable was referred to as maximum mouth opening (MMO).

### 2.6. Study Risk of Bias Assessment

The risk of bias in the source studies was assessed using RoB 2: A revised Cochrane risk-of-bias tool for randomized trials.

### 2.7. Effect Measures

To measure the effectiveness of injection treatment with intra-articular administration of NSAIDs, the difference in mean changes in the overall health-related quality of life, TMJ pain intensity, and range of mandibular abduction was calculated. The MedCalc software was used (version 22.023, MedCalc Software, Ostend, Belgium).

### 2.8. Synthesis Methods

Randomized clinical trials that successfully passed the selection and risk of bias assessment were included in the syntheses. First, numerical data were collected and tabulated. The results were then divided according to the domains assessed: (1) health-related quality of life, (2) articular pain, and (3) range of mandibular motion. Both outcomes from source reports (initial averages and averages after available observation periods) and outcomes obtained from own calculations (differences in averages for individual observation periods) were synthesized. The numerical data described above were visualized in charts. The Google Workspace software (version 2024-04-17, Google LLC, Mountain View, CA, USA) and MedCalc (version 22.023, MedCalc Software Ltd., Ostend, Belgium) were used.

### 2.9. Certainty Assessment

The quality of evidence was independently determined for each of the outcomes assessed. We included (1) the number of source reports, (2) the results of the risk of bias assessment for these studies, (3) the total size of the patient groups included in these studies, (4) the mean difference between study and control group, and (5) the scale effect expressed as Cohen’s d.

## 3. Results

### 3.1. Study Selection

**Figure 4:**
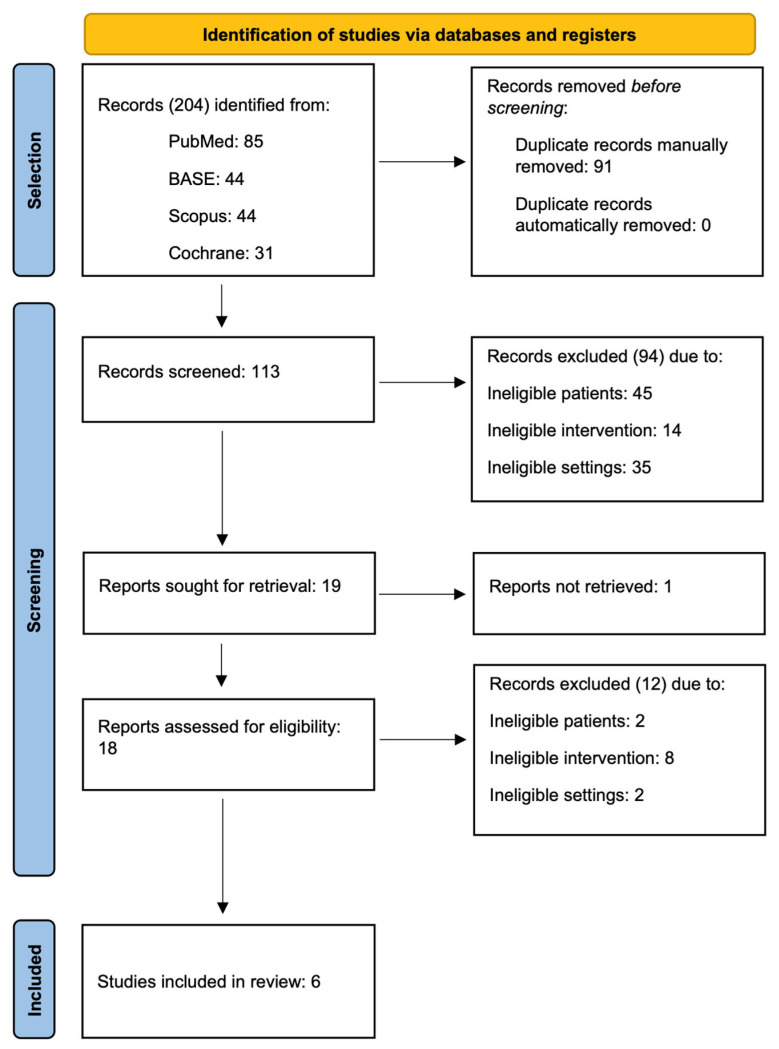
PRISMA flow diagram.

The search identified 204 records, of which 113 remained after manual deduplication (Figure 4). The initial selection with an inter-rater agreement of 92% (κ = 0.64) led to the exclusion of another 94 records. The report by Vallon et al. was not retrieved due to the lack of digitization of the paper article [34]. The remaining 18 reports were retrieved as a full text and assessed in detail for compliance with the established eligibility criteria. We tabulated the reports rejected at this stage, specifying the reasons for exclusion (Table A1). Ultimately, six randomized controlled trials were included.

### 3.2. Study Characteristics

The eligible studies were characterized by various detailed diagnoses for the included patients (Table 2). The number of patients in the research groups in the studies included in the review ranged from 7 to 25, and the number of patients in the control groups ranged from 11 to 75. The NSAIDs used by the researchers included diclofenac, piroxicam, and tenoxicam. Drug volumes administered during one intervention ranged from 1 to 2 mL; the only study that did not specify the drug dose was Yapici-Yavuz et al. [35]. The total number of interventions performed ranged from one to two. Follow-ups usually included a maximum period of 6 months, but an exception to this rule was the study by Raiesian et al. (1-week follow-up) and Gupta et al. (3-month follow-up) [36,37].

### 3.3. Risk of Bias in Studies

**Figure 5:**
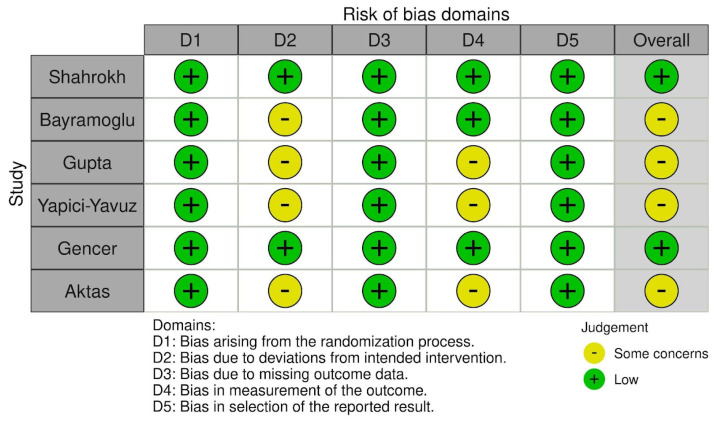
Risk of bias assessment—part 1.

**Figure 6:**
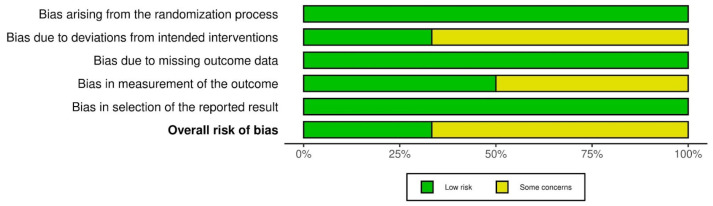
Risk of bias assessment—part 2.

The risk of bias was assessed using RoB 2: A revised Cochrane risk-of-bias tool for randomized controlled trials and is presented in Figure 5 and Figure 6.

### 3.4. Results of Individual Studies

Each of randomized control trials that were qualified for inclusion in this systematic review successfully passed the risk of bias assessment. Table 3 and Table 4 present numerical results extracted from the content of the report on these studies regarding temporomandibular joint pain and its changes (Table 3) and MMO and its changes (Table 4). For each value from the observation period, the mean difference was calculated relative to the base value of a given variable for a given group of patients.

### 3.5. Results of Syntheses

**Figure 7:**
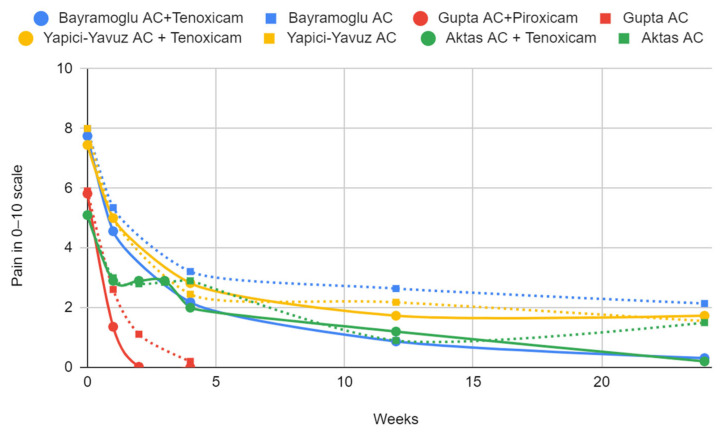
Pain change.

**Figure 8:**
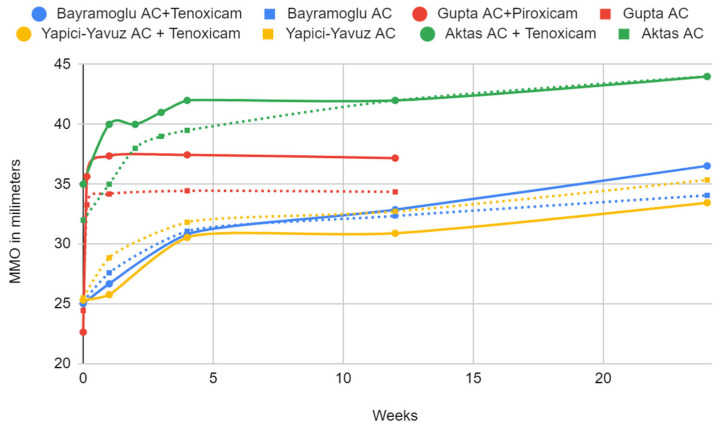
MMO change.

The available results are synthesized in Table 5 and Table 6 and presented in graphs (Figure 7 and Figure 8). The mean differences between the treated and placebo groups were calculated, along with the effect size. Statistically significant results are marked in the tables.

### 3.6. Certainty of Evidence

In randomized controlled trials assessing the difference between arthrocentesis alone and arthrocentesis supplemented with NSAID administration, subtle differences in joint pain intensity values were observed (Table 7). For tenoxicam, 4 weeks after the intervention, the differences did not exceed 1 point on a scale of 0–10. The results were contradictory regarding whether a better effect was achieved in the study or control group. In the case of piroxicam, no statistically significant difference was observed between the groups. At this stage, the average pain intensity was negligible in both groups of this study.

Concerning MMO, after 4 weeks of observation, treatment with tenoxicam did not result in statistically significant differences compared to arthrocentesis alone (Table 8). Piroxicam showed a statistically significant increase in MMO by almost 5 mm compared to the control group, with a large effect size. These results are based on only one trial with study and control groups of 11 patients each and some concerns regarding the risk of bias.

## 4. Discussion

Tenoxicam and piroxicam are the representatives of oxicams, which belong to NSAIDs. They inhibit both COX-1 and COX-2, thus providing therapeutic and side effects resulting from the properties of these enzymes [41,42]. These are inflammation and related symptoms’ (e.g., pain) suppression, gastrointestinal and kidney disorders, or development of hematological complications [43,44,45]. Piroxicam can be administered orally and intramuscularly and is characterized by a long half-life in the plasma. The drug penetrates the synovial fluid and joint spaces, which is beneficial in the context of articular symptoms treatment [46,47]. In the case of tenoxicam, the possible routes of administration are: oral, intravenous, intramuscular, and rectal [48,49]. In the treatment of joint diseases, drugs such as NSAIDs are used, but their prolonged intraoral administration is associated with an increasing risk of side effects’ development [50]. Administered intra-articularly, NSAIDs are expected to act locally and limit or eliminate systemic complications. Piroxicam and tenoxicam are known for eliminating joint pain; therefore, their local administration is examined, i.e., topical or intra-articular [51].

When selecting the method of drug administration, several factors should be taken into account, such as (1) the characteristics of the specific drug, (2) dose adjustment, (3) the administration route, (4) possible addition of other substances to increase the effectiveness of the active one, (5) systemic and local side effects, (6) pharmacokinetic features such as half-life, and (7) the ability to penetrate the selected tissues [52,53,54,55]. The multitude of combinations forces researchers to test those most likely to be effective in the first place [20,32]. Thanks to high-quality clinical trials and their syntheses, it is possible to focus future research on not only efficient but primarily safe solutions, which causes some of them to be gradually abandoned [56,57].

Based on an experimental rat study, Kim et al. discussed the intra-articular administration of piroxicam, emphasizing the concentration and retention in the synovial fluid. Cationic nanoparticle usage was proposed, which could extend the duration and effect of the drug in the joint while minimizing the risk of systemic side effects resulting from too rapid entry of piroxicam from the joint into the bloodstream. The concentration of piroxicam after injection with a cationic nanoparticle was higher than when using a drug solution [58]. Ishaq et al. compared the effects of triamcinolone (a glucocorticoid) and piroxicam administered intra-articularly to rats. The histopathological analysis of, among others, chondrocytes, periosteal fibrosis, and perichondrial fibrosis indicates the advantage of piroxicam over triamcinolone in the context of chondroprotection [59]. Another rat study by Park et al. showed better intra-articular than intramuscular piroxicam effectiveness [60].

### 4.1. General Interpretation of the Results

In the protocol comparing arthrocentesis followed by tenoxicam administration with joint lavage alone, repeated in three studies, no statistically significant differences in joint pain or the range of jaw abduction were observed, or they were subtle or contradictory [35,38,40]. Therefore, in the current state of knowledge, there is no justification for supplementing arthrocentesis with an injection of tenoxicam. The analogous administration of piroxicam showed promising results, albeit in only one study with a small sample size. This systematic review also identified the administration of tenoxicam and diclofenac without prior joint rinsing, showing promise for pain relief over the saline placebo [37].

### 4.2. Limitations of the Evidence

Despite searching numerous medical databases using broad-spectrum engines, a few clinical studies on the administration of NSAIDs into TMJ cavities were identified. Among these studies, only some had a control group and randomization, which limits the evidence. Eligible randomized controlled trials on the treatment of TMJs with intra-articular injections of NSAIDs are highly heterogeneous. The differences among these studies results, among others, from the type of injection protocol used (number of administrations, injectable volume, number of interventions), type of drug administered (tenoxicam, piroxicam, etc.), and co-interventions (non-surgical treatment, arthrocentesis) [35,36,37,38,39,40].

### 4.3. Limitations of the Review Processes

The query was prepared in English, which excluded papers without keywords, titles, or abstracts in this language. Studies involving more invasive interventions, i.e., intra-articular administration of NSAIDs during arthrocentesis, were excluded due to the desire to evaluate the treatment results quantitatively.

### 4.4. Implications of the Results

The combined treatment of temporomandibular disorders consists of many therapeutic interventions, including, but not limited to, psychotherapy, biofeedback, physiotherapy, pharmacotherapy, splint therapy, minimally invasive surgical interventions, including intra-articular injections, and arthroscopic and open surgery [20,27,28,29,61]. The selection of appropriate therapeutic methods depends on the clinical case and may be challenging [62]. The administration of drugs directly into the TMJ joint cavity seems tempting due to the expected local and rapid effect [56,57]. However, the intra-TMJ administration of corticosteroids, for example, turned out to be most strongly correlated with the occurrence of complications in injection treatment [57]. Moreover, the current systematic review did not demonstrate the validity of administering local anesthetics to the TMJs [56]. These emphasize the importance of assessing the feasibility of administering NSAIDs via the intra-articular route in clinical practice.

## 5. Conclusions

In the current state of knowledge, no conclusive scientific evidence supports the need to inject non-steroidal anti-inflammatory drugs into the temporomandibular joint cavities to relieve joint pain or increase the range of mandibular abduction. Preliminary reports on piroxicam with arthrocentesis and tenoxicam or diclofenac without rinsing warrant further research.

## Figures and Tables

**Table 1:** Eligibility criteria.

	Included	Excluded
Patients	TMJ disorders	Cadaver and animal studies
Intervention	Intra-articular NSAID injection	Arthroscopy and open surgery
Control	Placebo or other injectable	Not applicable
Outcomes	Quality of life, pain, or mandibular mobility	Non-quantitative results
Settings	Randomized controlled trials	Preprints, conference papers

**Table 2:** Study characteristics.

First Author, Publication Year	Digital Object Identifier (or PMID)	Diagnosis	Patients/TMJs in Study Group	Substance in Study Group, Concentration	Patients/TMJs in Control Group	Substance in Control Group	Dosage per Intervention	Interventions Number	Follow-Up
Bayramoglu, 2023 [38]	10.1186/s12903-023-02852-z	TMJ OA	16/N/S	AC + Tenoxicam, 10 mg/mL	14/N/S	AC	2 mL, 20 mg	1	6 months
Gupta, 2023 [37]	10.7759/cureus.34580	ADDwoR	11/N/S	AC + Piroxicam, 20 mg/mL	11/N/S	AC	1 mL, 20 mg	1	3 months
Yapici-Yavuz, 2018 [35]	10.4317/medoral.22237	Non-reducing TMJ disc displacement	11/N/S	AC + Tenoxicam, N/S	11 + 11 + 11/N/S	(1) AC, (2) AC + methyloprednisolone acetate, (3) AC + HA	N/S	1	6 months
Raiesian, 2016 [36]	10.22037/rrr.v1i3.10664	TMJ arthralgia	12/N/S	Diclofenac sodium, 25 mg/mL	12 + 12/N/S	(1) morphine, 10 mg/mL (2) 0.9% saline	2 mL, 50 mg	2	1 week
Gencer, 2014 [39]	10.1016/j.jcms.2014.01.041	TMD	25/N/S	Tenoxicam, 20 mg/mL	25 + 25 + 25/N/S	(1) HA 10 mg/mL (2) betamethasone 7 mg/mL (3) 0.9% saline	2 mL, 40 mg	1	6 weeks
Aktas, 2010 [40]	10.1016/j.ijom.2010.02.010	TMJ DDwoR	7/10	AC + Tenoxicam, N/S	14/14	AC	2 mL	1	6 months

AC—arthrocentesis; ADDwoR—anterior disc displacement without reduction; HA—hyaluronic acid; N/S—not specified; OA—osteoarthritis; TMD—temporomandibular disorders; TMJ—temporomandibular joint.

**Table 3:** Temporomandibular joint pain (first line) and temporomandibular joint pain change (second line) in 0–10 scale.

First Author, Publication Year	Patient Group	Sample Size	Baseline	1 Week	2 Weeks	3 Weeks	4 Weeks	6 Weeks	12 Weeks	24 Weeks
Bayramoglu, 2023 [38]	AC + Tenoxicam	16	7.75 ± 2.32	4.56 ± 3.20 −3.19 * ± 0.99	N/S	N/S	2.18 ± 1.52 −5.57 * ± 0.69	N/S	0.87 ± 1.08 −6.88 * ± 0.64	0.31 ± 0.49 −7.44 * ± 0.59
	AC	14	8.0 ± 1.88	5.35 ± 3.34 −2.66 * ± 1.02	N/S	N/S	3.21 ± 3.21 −4.79 * ± 0.99	N/S	2.64 ± 2.70 −5.36 * ± 0.88	2.14 ± 2.76 −5.86 * ± 0.89
Gupta, 2023 [37]	AC + Piroxicam	11	5.82 ± 1.33	1.36 ± 0.59 −4.46 * ± 0.44	0.02 ± 0.08 −5.80 * ± 0.40	N/S	0.00 ± 0.00 −5.82 * ± 0.40	N/S	N/S	N/S
	AC	11	5.91 ± 1.30	2.61 ± 0.39 −3.30 * ± 0.41	1.11 ± 0.73 −4.80 * ± 0.45	N/S	0.20 ± 0.31 −5.71 * ± 0.40	N/S	N/S	N/S
Vapici-Yavuz, 2018 [35]	AC	11	8.00 ± 2.00	5.00 ± 3.66 −3.00 * ± 1.26	N/S	N/S	2.45 ± 3.14 −5.56 * ± 1.12	N/S	2.18 ± 2.82 −5.82 * ± 1.04	1.55 ± 2.77 −6.45 * ± 1.03
Aktas, 2010 [40]	AC + HA	11	7.09 ± 2.98	5.45 ± 2.91 −1.64 † ± 1.26	N/S	N/S	2.09 ± 1.81 −5.00 * ± 1.05	N/S	2.09 ± 2.43 −5.00 * ± 1.16	0.55 ± 0.69 −6.54 * ± 0.92
	AC + methyloprednisolone	11	8.45 ± 1.92	5.18 ± 3.71 −3.27 * ± 1.26	N/S	N/S	2.55 ± 2.34 −5.90 * ± 0.91	N/S	3.45 ± 3.59 −5.00 * ± 1.23	2.00 ± 2.61 −6.45 * ± 0.98
	AC + Tenoxicam	11	7.45 ± 2.62	5.00 ± 3.97 −2.45 † ± 1.43	N/S	N/S	2.82 ± 3.28 −4.63 * ± 1.27	N/S	1.73 ± 3.35 −5.72 * ± 1.28	1.73 ± 2.97 −5.72 * ± 1.19
Raiesian, 2016 [36]	Diclofenac	12	8.00	6.00 2.00	N/S	N/S	N/S	N/S	N/S	N/S
	Morphine	12	9.00	4.00 5.00	N/S	N/S	N/S	N/S	N/S	N/S
	0.9% saline	12	7.80	7.20 0.60	N/S	N/S	N/S	N/S	N/S	N/S
Gencer, 2014 [39]	Tenoxicam	25	N/S	3.53 ± 1.10	N/S	N/S	N/S	5.86 ± 0.80	N/S	N/S
	HA	25	N/S	3.18 ± 0.90	N/S	N/S	N/S	3.41 ± 1.20	N/S	N/S
	Betamethasone	25	N/S	4.13 ± 1.50	N/S	N/S	N/S	4.51 ± 1.00	N/S	N/S
	0.9% saline	25	N/S	7.93 ± 1.20	N/S	N/S	N/S	8.16 ± 1.50	N/S	N/S
Aktas, 2010 [40]	AC + Tenoxicam	14	5.10	2.90 2.20	2.90 2.20	2.90 2.20	2.00 3.10	N/S	1.20 3.90	0.20 4.90
	AC	7	5.10	3.00 2.10	2.80 2.30	2.85 2.25	2.90 2.20	N/S	0.90 4.20	1.50 3.60

AC—arthrocentesis; HA—hyaluronic acid; N/S—not specified; *—statistically significant (*p* < 0.05); †—no statistical significance (*p* ≥ 0.05).

**Table 4:** MMO values (first line) and MMO change (second line), in millimeters.

First Author, Publication Year	Patient Group	Sample Size	Baseline	1 Day	1 Week	2 Weeks	3 Weeks	4 Weeks	12 Weeks	24 Weeks
Bayramoglu, 2023 [38]	AC + Tenoxicam	16	25.03 ± 6.72	N/S	26.68 ± 5.75 +1.65 † ± 2.21	N/S	N/S	30.81 ± 7.18 +5.78 * ± 2.46	32.87 ± 5.17 +7.84 * ± 2.12	36.53 ± 4.52 +11.50 * ± 2.03
	AC	14	25.17 ± 6.15	N/S	27.60 ± 5.51 +1.43 † ± 2.21	N/S	N/S	31.07 ± 5.09 +5.90 * ± 2.13	32.35 ± 4.49 +7.18 * ± 2.04	34.07 ± 5.12 +8.90 * ± 2.14
Gupta, 2023 [37]	AC + Piroxicam	11	22.64 ± 3.38	35.64 ± 1.91 +13.00 * ± 1.17	37.36 ± 4.57 +14.72 * ± 1.71	N/S	N/S	37.45 ± 4.50 +14.81 * ± 1.70	37.18 ± 4.64 +14.54 * ± 1.73	N/S
	AC	11	24.45 ± 0.93	33.27 ± 2.33 +8.82 * ± 0.76	34.18 ± 2.04 +9.73 * ± 0.68	N/S	N/S	34.45 ± 2.50 +10.00 * ± 0.80	34.36 ± 2.29 +9.91 * ± 0.75	N/S
Vapici-Yavuz, 2018 [35]	AC	11	25.50 ± 6.11	N/S	28.86 ± 7.40 +3.36 † ± 2.89	N/S	N/S	31.82 ± 7.37 +6.32 * ± 2.89	32.73 ± 5.75 +7.23 * ± 2.53	35.36 ± 7.35 +9.86 * ± 2.88
Aktas, 2010 [40]	AC + HA	11	25.59 ± 6.02	N/S	27.50 ± 5.25 +1.91 † ± 2.41	N/S	N/S	30.09 ± 4.99 +4.50 † ± 2.36	33.19 ± 5.30 +7.60 * ± 2.41	37.55 ± 5.73 +11.96 * ± 2.51
	AC + methyloprednisolone	11	27.36 ± 4.57	N/S	27.95 ± 4.80 +0.59 † ± 2.00	N/S	N/S	30.50 ± 4.78 +3.14 † ± 1.99	33.09 ± 5.82 +5.73 * ± 2.23	33.68 ± 4.81 +6.32 * ± 2.00
	AC + tenoxicam	11	25.32 ± 6.99	N/S	25.77 ± 6.52 +0.45 † ± 2.88	N/S	N/S	30.55 ± 8.20 +5.23 † ± 3.25	30.90 ± 6.53 +5.58 † ± 2.88	33.45 ± 7.59 +8.13 * ± 3.11
Raiesian, 2016 [36]	AC + Tenoxicam	14	35.00	N/S	40.00 +5.00	40.00 +5.00	41.00 +6.00	42.00 +7.00	42.00 +7.00	44.00 +9.00
	AC	7	32.00	N/S	35.00 +3.00	38.00 +6.00	39.00 +7.00	39.50 +7.50	42.00 +10.00	44.00 +12.00

AC—arthrocentesis; HA—hyaluronic acid; N/S—not specified; *—statistically significant (*p* < 0.05); †—no statistical significance (*p* ≥ 0.05).

**Table 5:** TMJ pain values (first line), TMJ pain changes (second line), mean differences, and effect sizes in AC + substance versus sole AC (placebo) comparison. Pain in 0–10 scale.

First Author, Publication Year	Patient Group	Sample Size	Baseline	1 Week	2 Weeks	3 Weeks	4 Weeks	6 Weeks	12 Weeks	24 Weeks
Bayramoglu, 2023 [38]	AC + Tenoxicam	16	7.75 ± 2.32	4.56 ± 3.20 −3.19 * ± 0.99	N/S	N/S	2.18 ± 1.52 −5.57 * ± 0.69	N/S	0.87 ± 1.08 −6.88 * ± 0.64	0.31 ± 0.49 −7.44 * ± 0.59
	AC	14	8.0 ± 1.88	5.35 ± 3.34 −2.66 * ± 1.02	N/S	N/S	3.21 ± 3.21 −4.79 * ± 0.99	N/S	2.64 ± 2.70 −5.36 * ± 0.88	2.14 ± 2.76 −5.86 * ± 0.89
	Mean difference			−0.53 † ± 0.37	N/S	N/S	−0.80 * ± 0.31	N/S	−1.52 * ± 0.28	−1.58 * ± 0.27
	95% confidence interval			−1.28–0.22	N/S	N/S	−1.43–−0.17	N/S	−2.09–−0.95	−2.14–−1.02
	Effect Size			−0.53 ± 0.37	N/S	N/S	−0.95 ± 0.39	N/S	−2.00 ± 0.45	−2.12 ± 0.46
	95% confidence interval			−1.26–0.20	N/S	N/S	−1.71–−0.19	N/S	−2.88–−1.12	−3.02–−1.23
Gupta, 2023 [37]	AC + Piroxicam	11	5.82 ± 1.33	1.36 ± 0.59 −4.46 * ± 0.44	0.02 ± 0.08 −5.80 * ± 0.40	N/S	0.00 ± 0.00 −5.82 * ± 0.40	N/S	N/S	N/S
	AC	11	5.91 ± 1.30	2.61 ± 0.39 −3.30 * ± 0.41	1.11 ± 0.73 −4.80 * ± 0.45	N/S	0.20 ± 0.31 −5.71 * ± 0.40 *	N/S	N/S	N/S
	Mean difference			−1.16 * ± 0.18	−1.00 * ± 0.18	N/S	−0.11 † ± 0.17	N/S	N/S	N/S
	95% confidence interval			−1.54–−0.78	−1.38–−0.62	N/S	−0.47–0.25	N/S	N/S	N/S
	Effect Size			−2.73 ± 0.59	−2.35 ± 0.55	N/S	−0.28 ± 0.43	N/S	N/S	N/S
	95% confidence interval			−3.89–−1.57	−3.44–−1.26	N/S	−1.11–0.56	N/S	N/S	N/S
Vapici-Yavuz, 2018 [35]	AC + Tenoxicam	11	7.45 ± 2.62	5.00 ± 3.97 −2.45 † ± 1.43	N/S	N/S	2.82 ± 3.28 −4.63 * ± 1.27	N/S	1.73 ± 3.35 −5.72 * ± 1.28	1.73 ± 2.97 −5.72 * ± 1.19
	AC	11	8.00 ± 2.00	5.00 ± 3.66 −3.00 * ± 1.26	N/S	N/S	2.45 ± 3.14 −5.56 * ± 1.12	N/S	2.18 ± 2.82 −5.82 * ± 1.04	1.55 ± 2.77 −6.45 * ± 1.03
	Mean difference			0.55 † ± 0.58	N/S	N/S	0.93 † ± 0.51	N/S	0.10 † ± 0.50	0.73 † ± 0.48
	95% confidence interval			−0.65–1.75	2.90 2.20	2.90 2.20	−0.14–2.00	N/S	−0.94–1.14	−0.26–1.72
	Effect Size			3.00 2.10	2.80 2.30	2.85 2.25	0.78 ± 0.44	N/S	0.90 4.20	1.50 3.60
	95% confidence interval						−0.09–1.64	N/S		
Aktas, 2010 [40]	AC + Tenoxicam	14	5.10				2.00 3.10	N/S		
	AC	7	5.10				2.90 2.20	N/S		
	Difference						0.90	N/S		

AC—arthrocentesis; N/S—not specified; *—statistically significant (*p* < 0.05); †—no statistical significance (*p* ≥ 0.05).

**Table 6:** MMO values (first line), MMO pain changes (second line), mean differences, and effect sizes in AC + substance versus sole AC (placebo) comparison. MMO in millimeters.

First Author, Publication Year	Patient Group	Sample Size	Baseline	1 Day	1 Week	2 Weeks	3 Weeks	4 Weeks	12 Weeks	24 Weeks
Bayramoglu, 2023 [38]	AC + Tenoxicam	16	25.03 ± 6.72	N/S	26.68 ± 5.75 +1.65 † ± 2.21	N/S	N/S	30.81 ± 7.18 +5.78 * ± 2.46	32.87 ± 5.17 +7.84 ± 2.12 *	36.53 ± 4.52 +11.50 ± 2.03 *
	AC	14	25.17 ± 6.15	N/S	27.60 ± 5.51 +1.43 † ± 2.21	N/S	N/S	31.07 ± 5.09 +5.90 * ± 2.13	32.35 ± 4.49 +7.18 * ± 2.04	34.07 ± 5.12 +8.90 * ± 2.14
	Mean difference			N/S	0.22 † ± 0.81	N/S	N/S	−0.12 † ± 0.85	0.66 † ± 0.76	2.60 * ± 0.76
	95% confidence interval			N/S	−1.44–1.87	N/S	N/S	−1.85–1.61	−0.90–2.22	1.04–4.16
	Effect Size			N/S	0.10 ± 0.37	N/S	N/S	−0.05 ± 0.37	0.32 ± 0.37	1.25 ± 0.40
	95% confidence interval			N/S	−0.62–0.82	N/S	N/S	−0.77–0.67	−0.40–1.04	0.47–2.03
Gupta, 2023 [37]	AC + Piroxicam	11	22.64 ± 3.38	35.64 ± 1.91 +13.00 * ± 1.17	37.36 ± 4.57 +14.72 * ± 1.71	N/S	N/S	37.45 ± 4.50 +14.81 * ± 1.70	37.18 ± 4.64 +14.54 * ± 1.73	N/S
	AC	11	24.45 ± 0.93	33.27 ± 2.33 +8.82 * ± 0.76	34.18 ± 2.04 +9.73 * ± 0.68	N/S	N/S	34.45 ± 2.50 +10.00 * ± 0.80	34.36 ± 2.29 +9.91 * ± 0.75	N/S
	Mean difference			4.18 * ± 0.42	4.99 * ± 0.56	N/S	N/S	4.81 * ± 0.57	4.63 * ± 0.57	N/S
	95% confidence interval			3.30–5.06	3.83–6.15	N/S	N/S	3.63–5.99	3.44–5.82	N/S
	Effect Size			4.24 ± 0.77	3.83 ± 0.72	N/S	N/S	3.62 ± 0.69	3.47 ± 0.68	N/S
	95% confidence interval			2.73–5.74	2.43–5.24	N/S	N/S	2.26–4.98	2.15–4.80	N/S
Vapici-Yavuz, 2018 [35]	AC + Tenoxicam	11	25.32 ± 6.99	N/S	25.77 ± 6.52 +0.45 † ± 2.88	N/S	N/S	30.55 ± 8.20 +5.23 † ± 3.25	30.90 ± 6.53 +5.58 † ± 2.88	33.45 ± 7.59 +8.13 * ± 3.11
	AC	11	25.50 ± 6.11	N/S	28.86 ± 7.40 +3.36 † ± 2.89	N/S	N/S	31.82 ± 7.37 +6.32 * ± 2.89	32.73 ± 5.75 +7.23 * ± 2.53	35.36 ± 7.35 +9.86 * ± 2.88
	Mean difference			N/S	−2.91 * ± 1.23	N/S	N/S	−1.09 † ± 1.31	−1.65 † ± 1.16	−1.73 † ± 1.28
	95% confidence interval			N/S	−5.48–−0.34	N/S	N/S	−3.83–1.65	−4.06–0.76	−4.40–0.94
	Effect Size			N/S	−1.01 ± 0.45	N/S	N/S	−0.35 ± 0.43	−0.61 ± 0.44	−0.58 ± 0.44
	95% confidence interval			N/S	−1.90–−0.12	N/S	N/S	−1.20–0.49	−1.46–0.25	−1.43–0.28
Aktas, 2010 [40]	AC + Tenoxicam	14	35.00	N/S	40.00 5.00	40.00 5.00	41.00 6.00	42.00 7.00	42.00 7.00	44.00 9.00
	AC	7	35.00	N/S	35.00 3.00	38.00 6.00	39.00 7.00	39.50 7.50	42.00 10.00	44.00 12.00
	Difference			N/S	2.00	−1.00	−1.00	−0.50	−3.00	−3.00

AC—arthrocentesis; N/S—not specified; *—statistically significant (*p* < 0.05); †—no statistical significance (*p* ≥ 0.05).

**Table 7:** Pain outcomes’ certainty of evidence.

	Number of Source Studies	Risk of Bias Assessment Results	Total Number of Patients in Study Groups	Total Number of Patients in Control Groups	Outcomes Mean Difference Range in 4 Weeks	Statistical Significance of Mean Differences	Effect Size Range in 4 Weeks
AC + Tenoxicam vs. AC	3	Some concerns	41	32	−0.80–0.93	Various	−0.95–0.78
AC + Piroxicam vs. AC	1	Some concerns	11	11	−0.11	*p* > 0.05	−0.28

**Table 8:** MMO outcomes’ certainty of evidence.

	Number of Source Studies	Risk of Bias Assessment Results	Total Number of Patients in Study Groups	Total Number of Patients in Control Groups	Outcomes Mean Difference Range in 4 Weeks	Statistical Significance of Mean Differences	Effect Size Range in 4 Weeks
AC + Tenoxicam vs. AC	3	Some concerns	41	32	−1.09–−0.12	*p* > 0.05	−0.05–−0.35
AC + Piroxicam vs. AC	1	Some concerns	11	11	4.81	*p* < 0.05	3.62

## Data Availability

All collected data are included in the content of this article. The protocol was not published prior to the publication of this review. PROSPERO registration number: CRD42024557962.

## Appendix A

**Table A1:** Studies rejected during the full-text evaluation.

First Author, Publication Year	Digital Object Identifier or PubMed Unique Identifier	Reason for Exclusion
Zhong, 2023 [63]	10.1016/j.mtbio.2023.100573	Ineligible patients
Gray, 1994 [64]	10.1038/sj.bdj.4808513	Ineligible settings
Ishida, 2003 [65]	10.1016/S0915-6992(03)80027-4	Ineligible intervention
Daif, 2012 [66]	10.1016/j.tripleo.2011.08.006	Ineligible intervention
Aktas, 2005 [67]	10.1016/s0901-5027(05)81355-4	Ineligible settings
Cen, 2022 [68]	10.1007/s00784-021-04241-8	Ineligible intervention
Tran, 2019 [69]	31553549	Ineligible intervention
Emes, 2014 [70]	10.1016/j.jcms.2013.06.010	Ineligible intervention
Lin, 1994 [71]	10.1016/s0901-5027(05)80038-4	Ineligible intervention
Lorber, 1974 [72]	4528866	Ineligible patients
Sinisalu, 2016	Not specified	Ineligible intervention
Dammling, 2022 [73]	10.21037/fomm-20-37	Ineligible intervention
